# DDX5 Can Act as a Transcription Factor Participating in the Formation of Chicken PGCs by Targeting *BMP4*

**DOI:** 10.3390/genes15070841

**Published:** 2024-06-26

**Authors:** Qisheng Zuo, Wei Gong, Zeling Yao, Kai Jin, Yingjie Niu, Yani Zhang, Bichun Li

**Affiliations:** 1 Key Laboratory of Animal Breeding Reproduction and Molecular Design for Jiangsu Province, College of Animal Science and Technology, Yangzhou University, Yangzhou 225009, China; mx120220894@stu.yzu.edu.cn (W.G.); 211603122@stu.yzu.edu.cn (Z.Y.); ynzhang@yzu.edu.cn (Y.Z.); zqs081901427@126.com (B.L.); 2 Joint International Research Laboratory of Agriculture and Agri-Product Safety of Ministry of Education of China, Yangzhou University, Yangzhou 225009, China; 007838@yzu.edu.cn (K.J.); niuyj@yzu.edu.cn (Y.N.)

**Keywords:** chicken, primordial germ cell, *DDX5*, transcription factor

## Abstract

As an RNA binding protein (RBP), DDX5 is widely involved in the regulation of various biological activities. While recent studies have confirmed that DDX5 can act as a transcriptional cofactor that is involved in the formation of gametes, few studies have investigated whether DDX5 can be used as a transcription factor to regulate the formation of primordial germ cells (PGCs). In this study, we found that *DDX5* was significantly up-regulated during chicken PGC formation. Under different PGC induction models, the overexpression of *DDX5* not only up-regulates PGC markers but also significantly improves the formation efficiency of primordial germ cell-like cells (PGCLC). Conversely, the inhibition of *DDX5* expression can significantly inhibit both the expression of PGC markers and PGCLC formation efficiency. The effect of *DDX5* on PGC formation in vivo was consistent with that seen in vitro. Interestingly, *DDX5* not only participates in the formation of PGCs but also positively regulates their migration and proliferation. In the process of studying the mechanism by which *DDX5* regulates PGC formation, we found that *DDX5* acts as a transcription factor to bind to the promoter region of *BMP4*—a key gene for PGC formation—and activates the expression of *BMP4*. In summary, we confirm that DDX5 can act as a positive transcription factor to regulate the formation of PGCs in chickens. The obtained results not only enhance our understanding of the way in which *DDX5* regulates the development of germ cells but also provide a new target for systematically optimizing the culture and induction system of PGCs in chickens in vitro.

## 1. Introduction

The unique migration of PCGs in the blood of poultry provides a new strategy for the conservation of species resources and the production of transgenic animals [1]. Over the past few years, an increasing number of studies have focused on the mechanisms regulating the formation of chicken PGCs [2,3], with the aim of constructing a chicken PGC formation regulatory network and optimizing in vitro culture conditions for the better application of PGCs.

In addition to key genes, a large number of transcription factors (TFs) with high functional conservation are involved in the formation of PGCs in different species. For example, BLIMP1 [4], as a key TF regulating the formation of PGCs, not only regulates the expression of target genes but may also affect epigenetic modification during the specialization of PGCs [5,6]. Studies have shown that the process of PGC formation in mice after *BLIMP1* mutation is abnormal, manifesting as a significant decrease in the number of PGCs in embryonic gonads [7]. Other TFs with similar functions include PRDM14 [8] and TCFAP2C [9]. It has been shown that BLIMP1, TCFAP2C, and PRDM14 can regulate epigenetic modification norms during PGC formation through an interaction system. Importantly, these three transcription factors (BLIMP1/TCFAP2C/PRDM14) can achieve PGC induction without the need for other cytokines in vitro [9,10]. As a pluripotent transcription factor, *OCT4* has an obvious dose-dependent effect on the formation of PGCs [11]. Studies have shown that the expression of *OCT4* can only be maintained in a small range during the regulation of PGC formation [12], as PGCs with *OCT4* over-expression or interference will lose their original biological characteristics. Of course, in addition to the above TFs, SOX family members [13], STAT1 [14], and so on also play important regulatory roles in the formation of PGCs, which means that the formation of PGCs cannot be separated from a huge regulatory network composed of TFs.

DEAD box helicase 5 (DDX5), an important RNA helicase, is mainly involved in the processes of RNA secondary structure unwinding, RNA transcription stabilization, and microRNA formation, among other processes [15]. Due to the presence of functional domains that bind DNA sequences in the structure of *DDX5*, it can be directly involved in regulating gene expression as a TF, although few relevant reports have been published to date. However, it is interesting to note that many studies have provided indirect evidence for the potential of DDX5 as a TF; for example, DDX5 can participate in the regulation of gene expression as a member of the transcriptional inhibition complex in Wnt signaling and of the transcriptional activation complex in Notch signaling [16,17]. It has been found that DDX5 can function as a transcription cofactor of MYOD and RUNX2 in myogenesis and skeletal development [18]. In the last several years, researchers have begun to confirm that *DDX5* can play a role in gametogenesis, especially in the further development of gonocytes [15]. Spermatogonia in the gonads of male mice are unable to undergo normal meiosis to form spermatozoa without *DDX5* [19]. Although related studies have shown that *DDX5* has little impact on the germ cells in early embryos, whether DDX5 functions as a transcription factor in this context has not yet been determined.

In a previous study, we determined that *DDX5* was significantly up-regulated during chicken PGC formation through RNA-seq. Furthermore, we confirmed that *DDX5* is involved in chicken PGC formation, as a regulatory factor regulating the expression of the key gene *BMP4*. Our results not only enrich knowledge of the biological function of *DDX5* but also provide a theoretical basis for further construction of the developmental regulatory network of chicken PGCs.

## 2. Materials and Methods

### 2.1. Cell Culture and PGCLC Induction

The blastocyst from fertilized eggs (stage X, E0) and the genital ridge tissues of chicken embryos at E4.5–E7.5 were used to isolate ESCs and PGCs, respectively [3]. Specific media compositions for ESCs and PGCs are detailed in Supplementary Table S2.

In our previous study, both one- and two-step models were established for the PGCLC induction process [3]. The two-step induction model specifically involves initially culturing ESCs in embryonic body (EB) induction medium for 4 days, followed by a transition to a PGCLC induction medium for a duration of 2 to 4 days. Alternatively, the one-step process simply entails culturing ESCs continuously in a PGCLC induction medium for a total of 8 days.

### 2.2. RNA Extraction, Purification, and Sequencing

The sex of ESCs and PGCs from different embryos was identified through PCR [20]. The total RNA of male and female ESCs and PGCs was extracted for library construction using TRNzol (DP424, Tiangen, Beijing, China). RNA purity and concentration were evaluated using a NanoDrop 2000 spectrophotometer (Thermo Scientific, Waltham, MA, USA). RNA integrity was assessed using an Agilent 2100 Bioanalyzer (Agilent Technologies, Santa Clara, CA, USA). The libraries were constructed with a VAHTS Universal V6 RNA-seq Library Prep Kit and sequenced on an Illumina Novaseq 6000 platform by Shanghai OE Biotechnology (Shanghai, China).

### 2.3. Differential Expression Analysis and Functional Enrichment

The raw data were quality-assessed and filtered using FastQC to obtain high-quality and relatively accurate valid data. Gene expression (FPKM value) was analyzed with Cuffdiff after comparing the valid data of the samples with the reference genome (Gallus gallus-5.0/galGal5) using HISAT2 [20], and differentially expressed genes (DEGs) were screened by *p* < 0.05 and |log2FC| > 1 [21]. The processes and methodologies of the GO [22] and KEGG [23] enrichment analyses used for the DEGs were drawn from relevant studies [24].

### 2.4. Construction of Plasmid

RNA was extracted from PGCs through TRIzol extraction and then reverse-transcribed into cDNA (JN0004, Baiao LaiBo, Beijing, China). Primers were designed according to the CDS sequence of *DDX5* (NM_204827.2) provided by the NCBI. The full length of *DDX5* was amplified and connected to the pcDNA3.1 vector to construct a *DDX5* over-expression vector (oe-*DDX5*). The interference primers were designed for the full-length CDS of *DDX5*, which was connected to the pGMLV-SC5 skeleton to construct a *DDX5* interference expression vector (sh-*DDX5*). oe-*DDX5* and sh-*DDX5* were transfected into DF-1 cells for 48 h, then the cell samples were collected for the extraction of RNA, and the vector activities of oe-*DDX5* and sh-*DDX5* were verified through qRT-PCR. The construction process of the *BMP4* promoter luciferase reporter vector was the same as above. The BMP4 knockout vector (KO-*BMP4*) was constructed by our team previously [25]. The relevant primers are listed in Supplementary Table S1.

### 2.5. qRT-PCR (Quantitative Reverse Transcription Polymerase Chain Reaction)

Cell samples at 0, 2, 4, and 6 d were collected from different PGCLC-induced models, as well as ESCs and PGCs. The expression levels of PGC markers (*DDX5*, *DDX4*, *DAZL*, and *STRA8*) and triblastic marker genes (*PAX6*, *EMOES*, and *VIMENT*) were evaluated, using β-actin as an internal reference. The PCR reaction program was carried out according to the instructions provided for the ChamQ Universal SYBR qPCR Master Mix (Vazyme, Nanjing, China, Q711). In detail, the reagent system (20 μL) for the qRT-PCR reaction consisted of 10 μL 2 × ChamQ Universal SYBR qPCR Master Mix, 0.6 μL each of upstream and downstream primers, 2 μL of cDNA, and 6.8 μL of ddH_2_O. The PCR cycle consisted of 30 cycles of 98 °C for 10 s, 49 °C for 5 s, and 72 °C for 30 s, followed by long-term storage at 4 °C. The relative expression levels of the genes were calculated using the 2-ΔΔCt method [26].

### 2.6. FC (Flow Cytometry) to Detect the Efficiency of PGCLC

The generation efficiency of PGCLC cells was determined through flow cytometry. Cell samples were collected from different induction models at 6 d (cells with oe-*DDX5* and sh-*DDX5* treatments) and were prepared in the form of cell suspensions. The samples were fixed with 4% paraformaldehyde at 25 °C for 10 min. After washing with PBS three times, the collected cells were permeated with 0.25% Triton-x100 at 25 °C for 5 min. The samples were then washed twice with PBS and blocked with 0.1% BSA for 30 min at 37 °C. After washing with PBS once again, the collected cells were incubated with the primary antibody DDX4 (1:100; ab13840, Abcam, Cambridge, UK) at 37 °C for 2 h. Then, the samples were washed three times with PBS and incubated with the second antibody—FITC Conjugated AffiniPure Mouse Anti-Rabbit IgG (H + L) (1:200, BM2012, Boster, Pleasanton, CA, USA)—at 37 °C for 1 h. After washing three times with PBS, the samples were collected, and the flow cytometry assay was conducted using BDFACSCanto II to count the fluorescence signals of the DDX4 in all collected samples. A total of 10,000 hemocytes were analyzed for each sample.

### 2.7. CHIP-qPCR

A CHIP-qPCR assay was performed to detect the enrichment of DDX5 in the *BMP4* promoter region of ESCs and PGCs, using the anti-DDX5 antibody (Abcam, ab126730, Cambridge, UK). We followed the protocol of the CHIP kit from Millipore (17-371RF) to collect the ESCs and PGCs, which were then re-suspended in 500 µL of 16% formaldehyde and cross-linked for 10 min at 22 °C. After that, we centrifuged the mixture at 1000 rpm for 5 min to discard the supernatant, then re-suspended the cell pellet in SDS cell lysis buffer. We used ultrasonication to break down the DNA and then added 900 µL of dilution buffer with protease inhibitor II. The mixture was divided into three groups: the IgG group, the positive control group, and the DDX5 antibody group. We followed the kit’s protocol to collect and purify the DNA from each group and performed quality control tests. We detected the enrichment of DDX5 in the *BMP4* promoter region through qPCR. The primers are listed in Supplementary Table S1.

### 2.8. Double Luciferase Report Detection

DF-1 cells with oe-*DDX5* and sh-*DDX5* treatments were co-transfected with a *BMP4*-promoter luciferase reporter plasmid (*BMP4*-Promoter) and pRL-SV40 at a mass ratio of 30:1. To ensure an accurate comparison, a negative control group was set up, in which the *BMP4*-Promoter plasmid was omitted. Transfection was carried out using Fugene, with a Fugene (V)/*BMP4*-Promoter (M) ratio of 3:1. The cells were collected and processed for dual-luciferase analysis after transfection for 48 h. Then, 70 μL of cell lysis solution was added to each tube, followed by gentle mixing. Subsequently, 70 μL of luciferase reagent was added to each well, again with gentle mixing. The plate was then placed in a luminometer (Tecan, Männedorf, Switzerland) to measure firefly luminescence, which served as an indicator of *BMP4* promoter activity. After the initial measurement, 70 μL of STOP solution was added to each well, followed by gentle mixing. The plate was once again placed in the luminometer to read the Renilla luminescence.

### 2.9. EdU Detection

A Cell-Light EdU Apollo567 In Vitro Kit (C10310-1, RiboBio, Guangzhou, China,) was used for measuring the proliferation levels of PGCs treated with oe-*DDX5* and sh-*DDX5*. Briefly, PGCs were inoculated in 24-well plates at a density of 1 × 10^5^ cells per well and were subsequently labeled using 50 μL of EdU medium containing 50 μM EdU. Following this step, the cells were immobilized with 4% paraformaldehyde. Subsequently, the Apollo staining procedure was conducted. The DNA was labeled with Hoechst33342, then the labeled cells were observed under a fluorescence microscope (Leica, Wetzlar, Germany, DMC6200).

### 2.10. Transwell Culture Assay

To assess the migratory capabilities of PGCs, a Transwell culture assay was conducted. A total of 24 plates were placed into the Transwell chamber, and 10^4^ PGCs under different treatments (including those with oe-*DDX5* and sh-*DDX5*) were collected and inoculated into the Transwell cells. After 48 h of culture, the Transwell chamber was removed to observe the number of cells in the 24 culture plates for statistical analysis.

### 2.11. PAS Staining

The cultured PGCs were collected and washed with PBS twice before suspending the cells in a volume ranging from 20 to 40 μL of fixation solution. The cells were spread evenly onto a microscope slide in a well-ventilated area. Following air-drying, they were gently washed with distilled water and allowed to dry naturally. Subsequently, 20 μL of Schiff’s reagent was applied in the dark and left for about 15 min of staining. After rinsing, first with an acidic solution and then with distilled water, to eliminate any excess stain, the slide was again allowed to dry naturally. Finally, the cells were covered with hematoxylin and stained for 2 min. The slide was rinsed thoroughly under running water before examination under a microscope (Leica, Wetzlar, Germany, DMC6200).

### 2.12. Data Analysis

All experiments were replicated at least 3 times, and the data are expressed as the mean ± SE. The data were sorted using Excel and then analyzed using GraphPad Prism 6 (GraphPad Software Inc., San Diego, CA, USA) for significance analysis and mapping. The significance differences between the data were obtained through *t*-tests and an ANOVA (* *p* < 0.05, significant difference; ** *p* < 0.01, extremely significant difference).

## 3. Results

### 3.1. The Expression of DDX5 Was Up-Regulated during the Formation of Chicken PGCs

**Figure 1:**
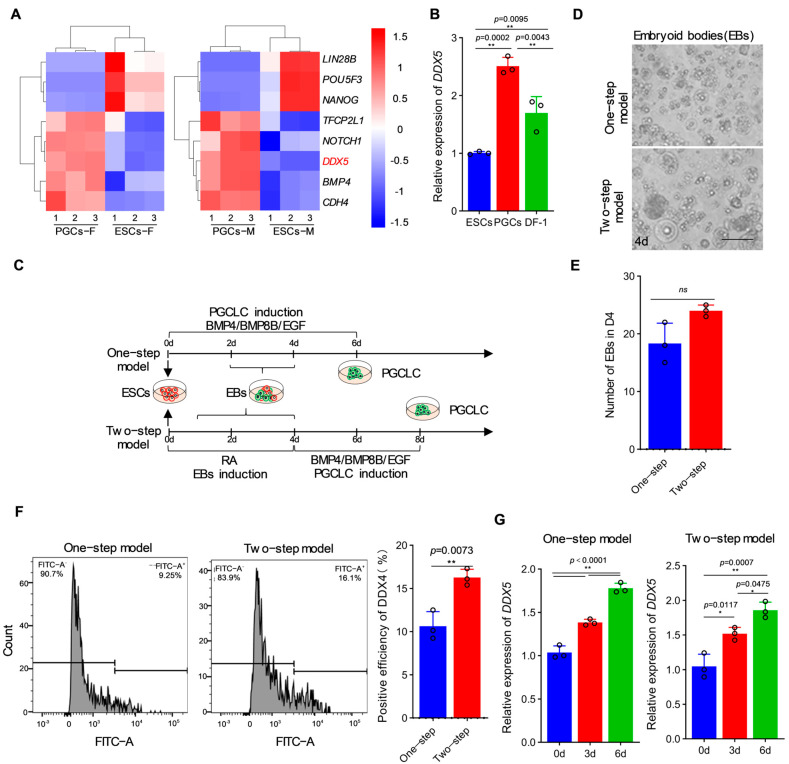
The expression of *DDX5* was up-regulated during the formation of chicken PGCs. (**A**) Transcriptional profiling of genes associated with pluripotency and germline development in chicken ESCs and PGCs. (**B**) Detection of *DDX5* expression in chicken ESCs, PGCs, and DF-1 (data are shown as mean ± SEM from *n* = 3 independent experiments, with a one-way ANOVA, ** *p* < 0.01: extremely significant difference). (**C**) Schematic diagram of the induction model of chicken PGCs in vitro. (**D**) EBs in the PGC induction model. Scale bar: 60 µm. (**E**) The number of EBs under two PGC induction models (data are shown as mean ± SEM, *n* = 3 independent experiments, with a *t*-test, ns: no significant difference). (**F**) Analysis of PGCLC formation efficiency under different induction models (data are shown as mean ± SEM, *n* = 3 independent experiments, with a *t*-test, ** *p* < 0.01: extremely significant difference). (**G**) Detection of DDX5 expression under different induction models (data are shown as mean ± SEM, *n* = 3 independent experiments, with a one-way ANOVA, * *p* < 0.05: significant difference; ** *p* < 0.01: extremely significant difference).

In order to study the dynamic changes in *DDX5* during the formation of chicken PGCs, we analyzed the RNA-seq data of male/female ESCs and PGCs (Supplementary Figure S1A–F). Among the obtained differentially expressed genes (DEGs), we found that during the formation of male/female PGCs, *NANOG* and *OCT4*, which are related to pluripotency, showed significant down-regulation (Figure 1A), while the PGC marker genes *BMP4*, *TFCP2L1*, and *CDH4* showed significant up-regulation (Figure 1A). These results were consistent with the basic characteristics of the samples. It is interesting to note that *DDX5* was significantly up-regulated during the differentiation of ESCs into PGCs, which finding was confirmed by qRT-PCR (Figure 1A,B). Based on these results, we can reasonably hypothesize that *DDX5* plays an important role in chicken PGC formation.

To further clarify the changes in *DDX5* during the formation of chicken PGCs, we detected the expression of germ cell markers and *DDX5* under different PGC induction models in vitro [3] (Figure 1C). In the one-step induction model, BMP4/BMP8B/EGF was used to induce PGCLC (Figure 1C), wherein the induction process lasted 4–6 days. The qRT-PCR results indicated that germ cell markers such as *DAZL*, *STRA8*, and *DDX4* were significantly up-regulated during induction, as well as *DDX5* (Supplementary Figure S2B,D). In the two-step induction model, in order to improve the formation efficiency of PGCLCs (Figure 1C), we first conducted RA induction in the ESCs for 4 days to cultivate a large number of EBs (Figure 1D,E), followed by induction using an induction medium (BMP4/BMP8B/EGF) for 2 days to obtain PGCLCs (Figure 1F). It was observed that the triblastoderm (*PAX6*, *EMOES*, and *VIMENT*) and germ cell markers were significantly up-regulated in both the one- and two-step induction models (Figure 1G, Supplementary Figure S2A–D). On the sixth day of induction, the expression of *DDX5* in the two-step induction model was significantly higher than that in the one-step induction model (Figure 1G). After combining the above results, we believe that *DDX5* is highly expressed during chicken PGC formation processes, both in vivo and in vitro, suggesting that it may be involved in the process of PGC formation.

### 3.2. DDX5 Significantly Improved the Formation Efficiency of PGCLC In Vitro

**Figure 2:**
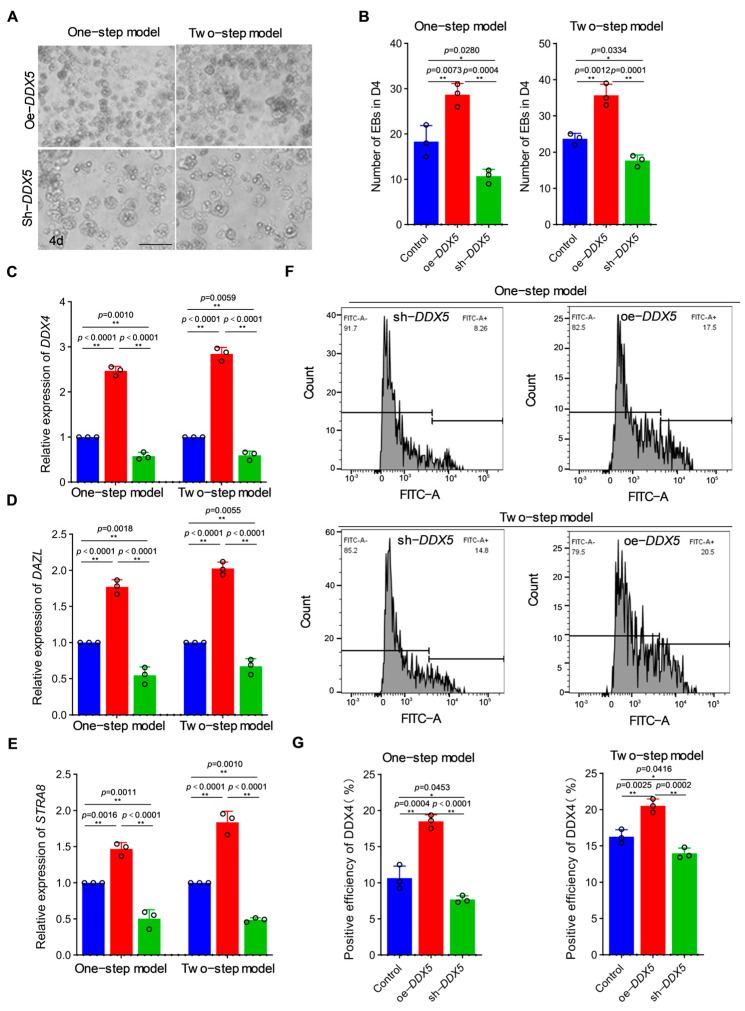
*DDX5* significantly improved the formation efficiency of PGCLC in vitro. (**A**) The effects of over-expressing and interfering with *DDX5* on EBs. Scale bar: 60 µm. (**B**) Statistical analysis of the EB number (data are shown as mean ± SEM, *n* = 3 independent experiments, and a one-way ANOVA, * *p* < 0.05: significant difference; ** *p* < 0.01: extremely significant difference). (**C**–**E**) Effect of *DDX5* on germ cell marker (*DAZL*, *DDX4*, and *STRA8*) expression in the induction model (data are shown as mean ± SEM, *n* = 3 independent experiments, and a one-way ANOVA, ** *p* < 0.01: extremely significant difference). (**F**,**G**) The effect of *DDX5* on PGCLC formation efficiency was analyzed by flow cytometry in different induction models (data are shown as mean ± SEM, *n* = 3 independent experiments, and a one-way ANOVA, * *p* < 0.05: significant difference; ** *p* < 0.01: extremely significant difference).

To study the function of *DDX5* in PGC formation systematically, active *DDX5* over-expression and interference expression vectors were constructed to transfect the ESCs in the induction models (Supplementary Figure S3). Morphological observations showed that both the induction models treated with oe-*DDX5* had an increased number of EBs, while treatment with sh-*DDX5* significantly decreased the number of EBs (Figure 2A,B). The results of qRT-PCR showed that germ cell markers (*DAZL*, *DDX4*, and *STRA8*) were significantly up-regulated after the over-expression of *DDX5* in the two induction models (at 6 d); conversely, these genes were significantly down-regulated after interference by *DDX5* (Figure 2C–E). Flow cytometry analysis of both the induction models on day 6 revealed that the over-expression of *DDX5* could significantly promote the formation efficiency of PGCLCs, while the inhibition of *DDX5* significantly reduced the formation efficiency of PGCs (Figure 2F,G). Based on these results, we initially concluded that *DDX5* could promote the differentiation of ESCs into PGCLCs in vitro.

### 3.3. DDX5 Has the Ability to Regulate the Migration and Proliferation of PGC

**Figure 3:**
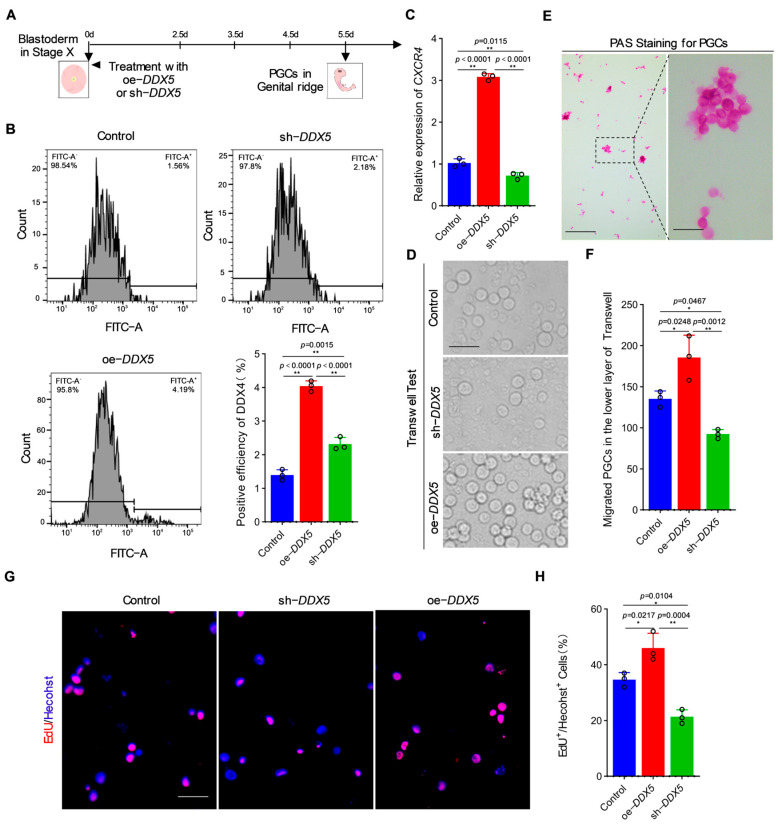
*DDX5* has the ability to regulate the migration and proliferation of PGCs. (**A**) Schematic diagram of *DDX5* modulating PGC formation in vivo. (**B**) Quantitative analysis of PGCs in the genital ridge under oe-*DDX5*/sh-*DDX5* treatments (data are shown as mean ± SEM, *n* = 3 independent experiments, and a one-way ANOVA, ** *p* < 0.01: extremely significant difference). (**C**) Effects of *DDX5* on the expression of migration-related genes (*CXCR4*) (data are shown as mean ± SEM, *n* = 3 independent experiments, and a one-way ANOVA, ** *p* < 0.01: extremely significant difference). (**D**–**F**) The effects of *DDX5* on the migration ability of PGCs were detected through a Transwell test. Scale bar: 60 µm (data are shown as mean ± SEM, *n* = 3 independent experiments, and a one-way ANOVA, * *p* < 0.05: significant difference; ** *p* < 0.01: extremely significant difference). (**G**,**H**) The EdU test was conducted to detect the effect of *DDX5* on the proliferation ability of PGCs. Scale bar: 60 µm (data are shown as mean ± SEM, *n* = 3 independent experiments, and a one-way ANOVA, * *p* < 0.05: significant difference; ** *p* < 0.01: extremely significant difference).

After clarifying the impact of *DDX5* on the formation of PGC in vitro, we injected *DDX5* over-expression and interference vectors into 2.5 d chicken embryos to explore the effects of *DDX5* on PGC formation in vivo (Figure 3A). The flow cytometric analysis was performed on the genital ridge of 5.5d chicken embryos from different treatment groups (oe-*DDX5* or sh-*DDX5*) and showed that the number of PGCs was significantly up-regulated in the genital ridge with oe-*DDX5* treatment, while the number of PGCs was significantly down-regulated in the genital ridge with sh-*DDX5* treatment (Figure 3B,C). These results suggest that *DDX5* also promotes PGC formation in vivo. Given that PGCs need to migrate through the vasculature from their originating position during the embryonic development process, the influence of *DDX5* on the migration and proliferation abilities of PGCs can reasonably explain this phenomenon. To confirm this hypothesis, we detected the expression of the migration marker gene *CXCR4* in PGCs with oe-*DDX5* or sh-*DDX5* treatment. The results showed that the over-expression of DDX5 could promote the expression of *Cxcr4*, while interference with *DDX5* could inhibit it (Figure 3C). To further clarify the effect of *DDX5* on the migration ability of PGCs, we cultured oe-*DDX5* and sh-*DDX5* PGCs in Transwells and found that after 48 h, the number of cells that had migrated to the bottom of the Transwell culture plate significantly increased after over-expressing *DDX5*, while the number of cells that had migrated after knocking down *DDX5* significantly decreased (Figure 3D–F). Meanwhile, we also detected the proliferation ability of oe-*DDX5* and sh-*DDX5* PGCs by EDU, and the results showed that the proliferation ability of PGC after the over-expression of *DDX5* was significantly enhanced, while the proliferation ability of PGCs after interference was significantly down-regulated (Figure 3G,H). All these results suggest that *DDX5* is involved in PGC formation by regulating the migration and proliferation of PGC.

### 3.4. DDX5 Participates in the Formation of PGCs by Regulating BMP4

**Figure 4:**
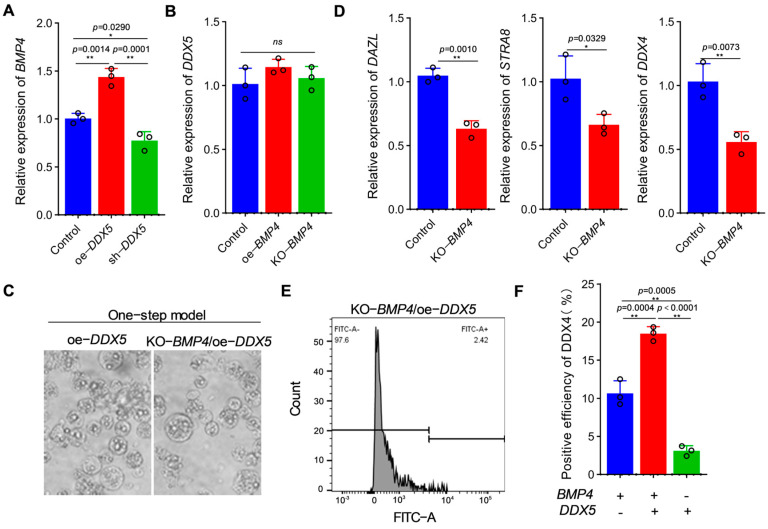
*DDX5* participates in the formation of PGCs by regulating *BMP4*. (**A**) The effect of *DDX5* on the expression of *BMP4* was detected via qRT-PCR (data are shown as mean ± SEM, *n* = 3 independent experiments, and a one-way ANOVA, * *p* < 0.05: significant difference; ** *p* < 0.01: extremely significant difference). (**B**) The effect of *BMP4* on the expression of *DDX5* was detected via qRT-PCR (data are shown as mean ± SEM, *n* = 3 independent experiments, and a one-way ANOVA, ns: no significant difference). (**C**) Effect of KO-BMP4 combined with oe-*DDX5* on the formation of EBs in a PGC-induced model. Scale bar: 60 µm. (**D**) Effect of KO-*BMP4* combined with oe-*DDX5* on PGC marker genes in a PGC induction model (data are shown as mean ± SEM, *n* = 3 independent experiments, and a *t*-test, * *p* < 0.05: significant difference; ** *p* < 0.01: extremely significant difference). (**E**,**F**) Effect of KO-*BMP4* combined with oe-*DDX5* on PGCLC efficiency (data are shown as mean ± SEM, *n* = 3 independent experiments, and a one-way ANOVA, ** *p* < 0.01: extremely significant difference).

It is well-known that the BMP4 signaling pathway is a crucial factor for the origin and migration of PGCs, and studies have shown that DDX5 is an important activator of BMP signaling. Therefore, we wondered whether there exists a regulatory relationship between *DDX5* and *BMP4.* We first detected the expression of *BMP4* in chicken DF-1 under oe-*DDX5* and sh-*DDX5* treatments (Figure 4A). The results indicated that the over-expression of *DDX5* can induce the expression of *BMP4*; however, the expression of *BMP4* was down-regulated after interference. Interestingly, we found that when over-expressing and knocking out *BMP4* in DF-1 cells, there was no significant change in the *DDX5* level (Figure 4B). Based on these results, we preliminarily concluded that *DDX5* is an upstream regulatory factor of *BMP4*; therefore, we speculated that *DDX5* may participate in the formation of PGCs by regulating *BMP4.* To prove this hypothesis, we modified the one-step PGC induction model (kd-*BMP4* and oe-*DDX5*), and found that, compared with the one-step induction model, the marker genes *DAZL*, *STRA8*, and *DDX4* were significantly down-regulated (Figure 4C,D). Importantly, the formation efficiency of PGCs was significantly reduced, as was consistent with our prediction (Figure 4E,F), which demonstrates that the over-expression of *DDX5* could not counteract the effect of *BMP4* knockout on PGC formation.

### 3.5. BMP4 Is an Important Target of DDX5 during the Formation of Chicken PGCs

**Figure 5:**
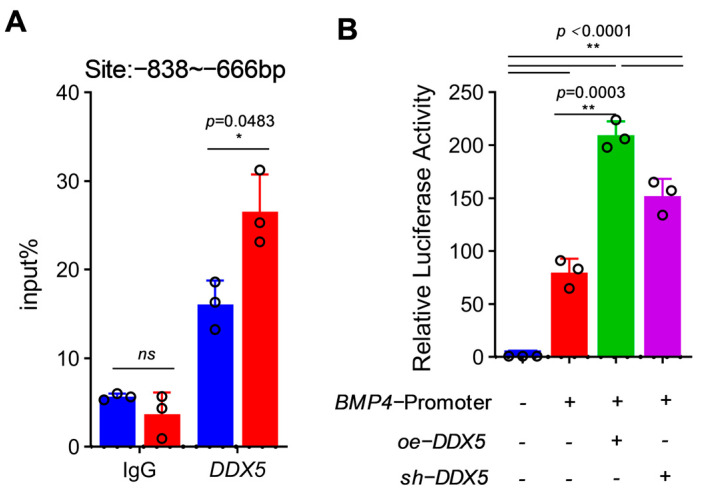
*BMP4* is an important target of *DDX5* during the formation of chicken PGCs. (**A**) Detection of *DDX5* enrichment level in the *BMP4* promoter region (data are shown as mean ± SEM, *n* = 3 independent experiments, and a *t*-test, * *p* < 0.05: significant difference; ns: no significant difference). (**B**) The effect of *DDX5* on *BMP4* promoter activity was detected using the dual-luciferase reporting system (data are shown as mean ± SEM, *n* = 3 independent experiments, and a one-way ANOVA, ** *p* < 0.01: extremely significant difference).

Studies have shown that *DDX5* can act as a co-activator that is involved in the transcriptional process of target genes. Therefore, we hypothesized that *DDX5* functions as a transcription factor in the regulation of *BMP4* gene expression. To verify this hypothesis, we first performed CHIP-qPCR detection, which revealed that *DDX5* was enriched in the *BMP4* promoter region, with a significantly higher level in PGCs than ESCs, suggesting that *DDX5* could bind to the *BMP4* promoter region (Figure 5A). In order to further clarify the regulatory relationship between *DDX5* and *BMP4*, we cloned the *BMP4* promoter into the pGL3.0-basic vector and detected the activity of the *BMP4* promoter using the dual-luciferase reporting system under oe-*DDX5* and sh-*DDX5* treatments. As expected, the activity of the *BMP4* promoter was significantly up-regulated after *DDX5* over-expression, while the activity of the *BMP4* promoter was significantly down-regulated after interference with *DDX5*, which is consistent with the observed effects of *DDX5* on *BMP4* expression levels (Figure 5B).

## 4. Discussion

In our study, we confirmed that *DDX5* acts as a positive regulator in the formation of chicken PGCs. Further studies revealed that *DDX5* can act as a transcription factor to regulate the expression of a target gene, *BMP4*, which is involved in the PCG formation process.

Both *DDX4* and *DDX5* belong to the DEAD-box helicase (Ddx) family. Studies have shown that DDX4 is widely involved in the formation of germ cells in different species and, so, can be used to trace the migration process of PGCs. The destruction of DDX4 expression will seriously affect the development of germ cells, even affecting the reproductive ability of individuals [27]. *DDX5* and *DDX4* share limited similarities. Studies have shown that *DDX5* has significant effects on germ cell development in different species. In mammals, *DDX5* has little effect on early male germ cells but does affect sperm formation. In zebrafish, it had no effect on the development of male germ cells and gametes but had a great effect on the development of ovaries and ova [15,19,28].

At present, there is no clear evidence that *DDX5* participates in the formation of chicken germ cells. However, it has been confirmed that *DDX5* can interact with β-catenin and activate the Wnt signaling pathway to regulate the expression of target genes [16,17]. Combined with our previous research results, as activated Wnt signaling normally regulates the formation process of chicken PGCs, we can preliminarily assume that DDX5 may participate in the development process of chicken germ cells [2]. Our study also showed that up-regulated *DDX5* expression during the formation of PGCs in chickens, as well as the deletion of *DDX5*, affected the formation of PGCs, both in vivo and in vitro, further confirming that *DDX5* potentially participates in the early formation of PGCs in chickens.

Many studies have found that DDX5 can be used as an RBP to participate in the post-transcriptional regulation of genes [29]; however, in recent years, more and more studies have confirmed that DDX5 can be bound to DNA sequences to participate in DNA transcription processes. Legrand reported that DDX5 binds to PLZF as a transcriptional co-activator that jointly regulates specific target genes involved in the formation of male germ cells [19]. Shuibin et al. showed that DDX5, as a component of the MAML1 protein complex in the Notch1 signaling pathway, can participate in the proliferation and apoptosis of leukemia cells [30]. Taken together, these studies demonstrate that DDX5 has the potential to act as a transcription factor regulating gene expression. In particular, our study confirmed that *DDX5* is involved in PGC formation by regulating the expression of BMP4—a key gene in PGC formation.

## Data Availability

The data used to support the findings of this study are included in the article. All details and materials of the experimental process can be obtained by contacting the corresponding author (006664@yzu.edu.cn). As we only selected some of the differential genes in the transcriptome data of male and female ESCs and PGCs in the manuscript, and since these transcriptome data are crucial in another study, the data used in the current study are not publicly available but are available from the corresponding author on reasonable request.

## Supplementary Materials

The following supporting information can be downloaded at: https://www.mdpi.com/article/10.3390/genes15070841/s1, Figure S1: Transcriptome data analysis of ESCs and PGCs (male and female). A,D: PCA analysis of ESCs and PGCs transcriptome data (male and female). B,E: Differential gene statistics between ESCs and PGCs (male and female). C,F: Volcanic map of differentially expressed genes between ESCs and PGCs (male and female); Figure S2: Analysis of gene expression in different PGC induction models. A,C: Expression of EB-related genes in two induction models. B,D: Expression of PGC-related genes in two induction models; Figure S3: Activity detection of DDX5 over-expression and interference expression vectors; Table S1: Sequence of related primers; Table S2: Cell culture medium composition.
