# Supplementary material for: DDX5 Can Act as a Transcription Factor Participating in the Formation of Chicken PGCs by Targeting BMP4
Source: Genes (Basel). 2024 Jun 26;15(7):841. doi: 10.3390/genes15070841 (PMC11276195; doi:10.3390/genes15070841)
Supplement: Supplementary file 1 [file genes-15-00841-s001.zip › Table S1 Sequence of related primers.pdf]

Table S1. Sequence of related primers

	F (5'-3')	R (5'-3')	usage
Ddx5	GATCGGGACCGTCTCGCCCA	ACTGAGACATTTAAATACAC	oe-Ddx5
Ddx5	TATTCAAGCACAAGGATGGC	CAGCAGCTACCTGCTGCACT	qRT-PCT
Ddx4	AGCACAGGTGGTGAACGAA	TGCTGGTGGATGGTAGGTT	qRT-PCT
Dazl	TACCCATTCGTCAACAACC	CACCTCCTTCACAGTACCATA	qRT-PCT
Stra8	CCACGGCTATTTCACACCTCTG	GCTCTTGGCAAGCATCCGTA	qRT-PCT
Pax6	CCGACGGGATGTACGACAAG	CATCTGGGCCTCATCGGAAT	qRT-PCT
Emoes	ACAACGGTGAGAGAACCGTC	CTGCTGCACAGGAGTAACGA	qRT-PCT
Viment	GGACCTGCTGAATGTAAAGA	AGGTTGGAATAGGCATGTTA	qRT-PCT
Cxcr4	ACTTGTCTTGCCTGGTCTA	TGATGCCAATGTAATACGG	qRT-PCT
Bmp4	AGTGATGAAGCCGCTGTCG	GCCCTGATGAGTCTGTGCC	qRT-PCT
sh-Ddx5	GGATCCGCAGTACAGATCAAGCAAAGACTCGAGTCTTTGCTTGATC TGTACTGCTTTTTGAATTC		sh-Ddx5
Bmp4-Promoter	GGAAGAGCGGCGCTGCGTTGGC	TCTCCCGGCCCCGCAGTCA	Bmp4-promoter
Bmp4-Promoter	TCTATGGGTGGTGGTTTGG	GAGAGGGCAGCTCCTGGGG	CHIP-qPCR
β-actin	ACCGCAAATGCTTCTAAAC	GACTGCTGCTGACACCTTC	qRT-PCT
