# Supplementary material for: DDX5 Can Act as a Transcription Factor Participating in the Formation of Chicken PGCs by Targeting BMP4
Source: Genes (Basel). 2024 Jun 26;15(7):841. doi: 10.3390/genes15070841 (PMC11276195; doi:10.3390/genes15070841)
Supplement: Supplementary file 1 [file genes-15-00841-s001.zip › Table S2 medium composition of cell culture.pdf]

Table S2. medium composition of cell culture.

	ESCs	PGCs	PGCLC	EB
1	KO-DMEM	DMEM	KO-DMEM	KO-DMEM
2	10% KSR	10% KSR	10% KSR	10% KSR
3	2.0 mM GlutaMax	2.0 mM GlutaMax	2.0 mM GlutaMax	2.0 mM GlutaMax
4	1% Non-EAA	1% Non-EAA	1% Non-EAA	1% Non-EAA
5	1% Chicken serum	1% Chicken serum	1% Chicken serum	1% Chicken serum
6	0.1 mM β-mercaptoethanol	0.1 mM β-mercaptoethanol	0.1 mM β-mercaptoethanol	0.1 mM β-mercaptoethanol
7	1% Penicillin-Streptomycin	1% Penicillin-Streptomycin	1% Penicillin-Streptomycin	1% Penicillin-Streptomycin
8	10 ng/mL LIF	0.2% ovalbumln	40 ng/mL BMP4	10^{-5} M RA
9	10 ng/mL bFGF	1.2mM sodium pyruvate	40 ng/mL BMP8b	
10	10 ng/mL hSCF	0.01% heparin	40 ng/mL EGF	
11	3 µM PD0325901	25ng/mL Activin A		
12	10 µM SB431542			
