# Supplementary figures and images for: DDX5 Can Act as a Transcription Factor Participating in the Formation of Chicken PGCs by Targeting BMP4
Source: Genes (Basel). 2024 Jun 26;15(7):841. doi: 10.3390/genes15070841 (PMC11276195; doi:10.3390/genes15070841)

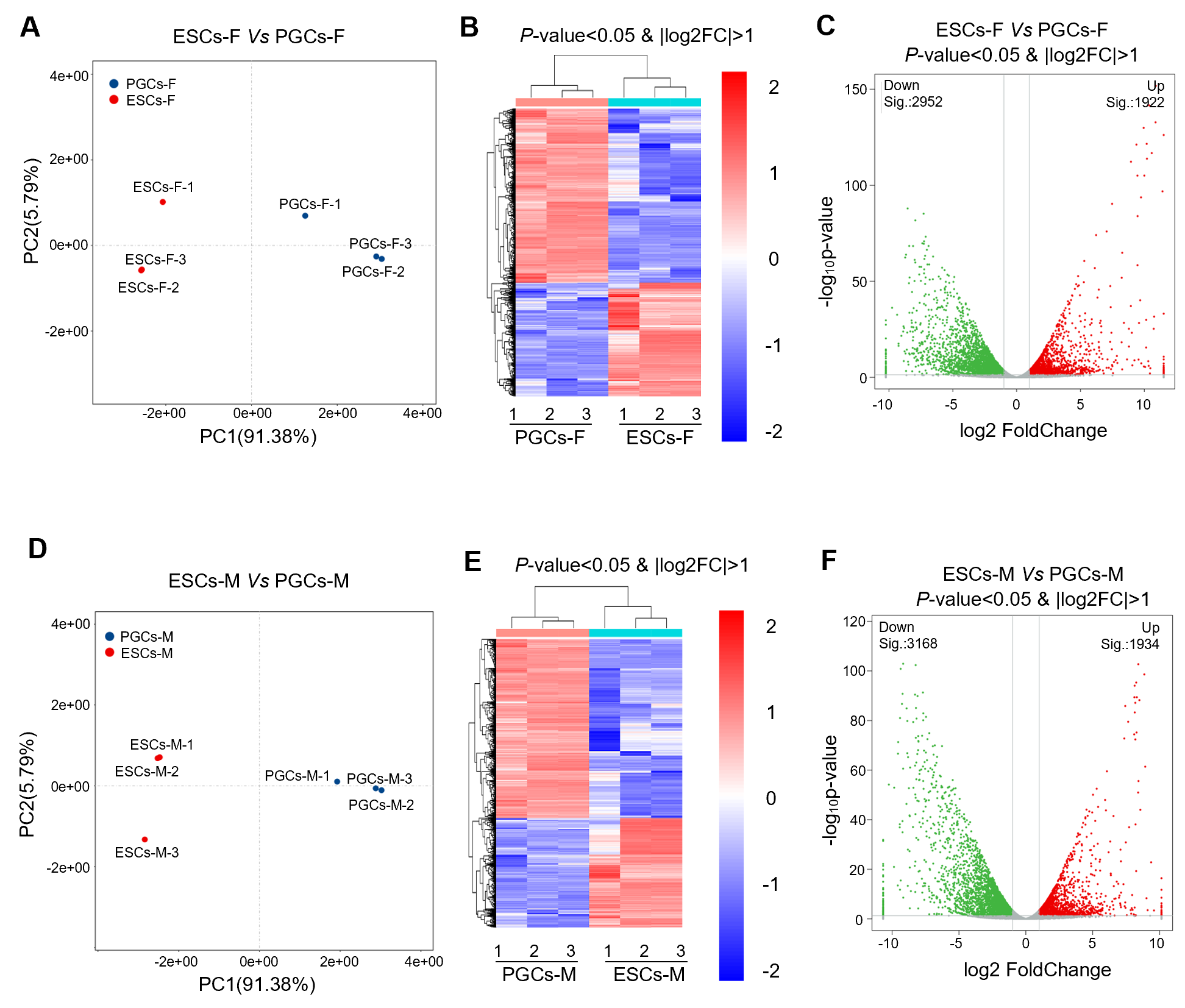

Supplement: Supplementary file 1 [file genes-15-00841-s001.zip › Fig S1.tif]

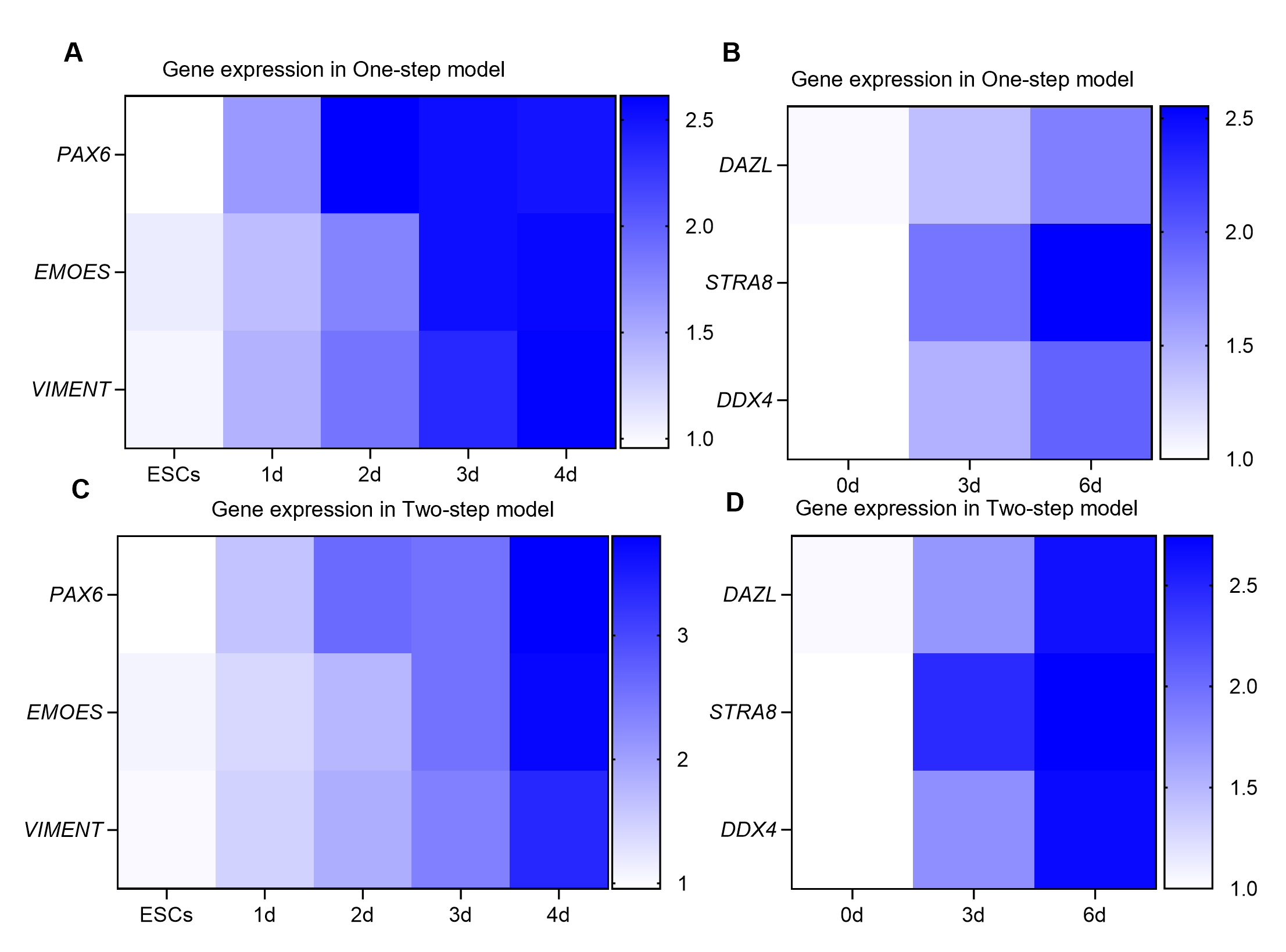

Supplement: Supplementary file 1 [file genes-15-00841-s001.zip › Fig S2.tif]

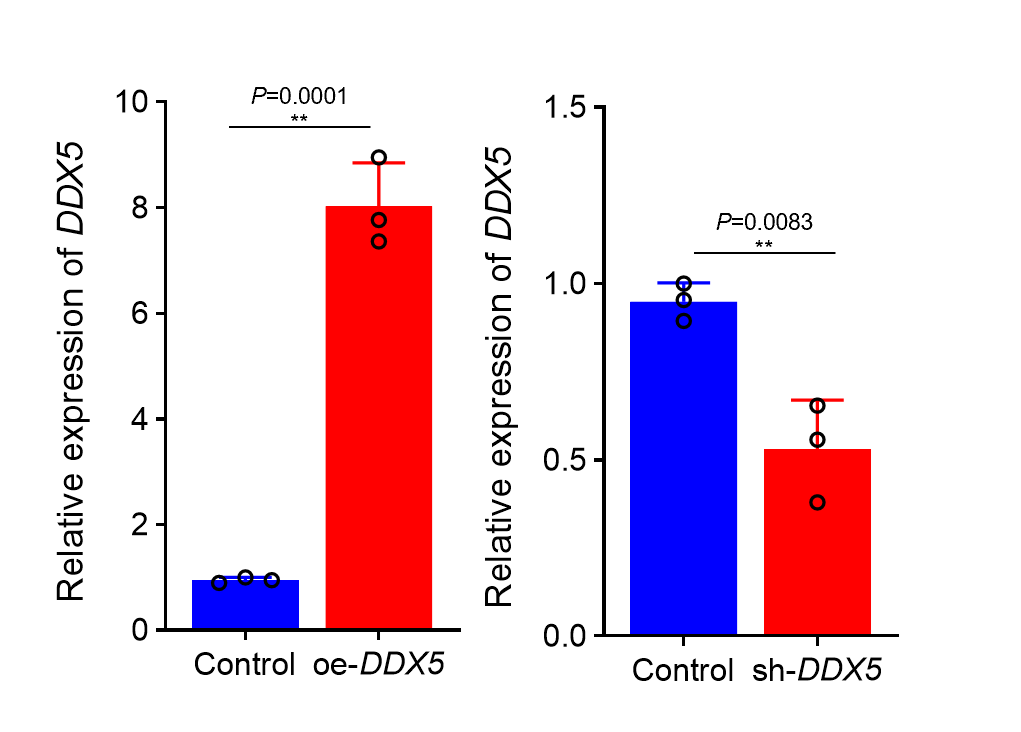

Supplement: Supplementary file 1 [file genes-15-00841-s001.zip › Fig S3.tif]
